# Integrating China in the international consortium for personalised medicine. a position paper on healthcare professionals’ education and citizens’ empowerment in personalised medicine

**DOI:** 10.1186/s12909-023-04420-z

**Published:** 2023-06-14

**Authors:** Flavia Beccia, Francesco Andrea Causio, Ilda Hoxhaj, Hui-Yao Huang, Lily Wang, Wenya Wang, Sara Farina, Tommaso Osti, Cosimo Savoia, Chiara Cadeddu, Walter Ricciardi, Stefania Boccia

**Affiliations:** 1 Section of Hygiene, University Department of Life Sciences and Public Health, Università Cattolica del Sacro Cuore, Largo Francesco Vito, 1 Rome, Rome, 00168 Italy; 2 Clinical Trials Center of National Cancer Center, Beijing, China; 3 BGI-Shenzhen, Shenzhen, China; 4 Center of Biotherapy, Beijing Tsinghua Changgang Hospital, Beijing, China; 5 Department of Woman and Child Health and Public Health, Fondazione Policlinico Universitario A. Gemelli IRCCS, Rome, 00168 Italy

**Keywords:** Personalised medicine, Citizen empowerment, Healthcare professionals, European Union, China

## Abstract

**Background:**

Personalised medicine (PM) has been fostered by technological and medical advances, but all stakeholders, including healthcare professionals, citizens and policy makers, should achieve adequate health literacy to promote PM implementation. The “Integrating China in the International Consortium for Personalised Medicine” (IC2PerMed) project, funded by the International Consortium for Personalised Medicine, focuses on this issue by highlighting the need to educate healthcare professionals and empower citizens. Within the aforementioned project, building on a mapping of European and Chinese policies in PM, experts in the field of PM participated in an online workshop and a following two-round Delphi survey, in order to identify the priority areas of intervention for healthcare professionals’ education and curricula, engagement and empowerment of citizens and patients.

**Results:**

Nine experts completed the survey and reached a consensus on seventeen priorities: seven were related to health professionals’ education and curricula, whereas ten on citizen and patients’ awareness and empowerment.

**Conclusion:**

These priorities emphasized the importance of education and health literacy, multidisciplinary and international collaboration, public trust, and consideration of ethical, legal, and social issues. The present experience highlights the relevance of the involvement of stakeholders in informing decision-makers, developing appropriate national plans, strategies, and policies, and ensuring the adequate implementation of PM in health systems.

## Background

Over the last two decades, although Personalised Medicine (PM) has revolutionized healthcare, tailoring prevention, diagnosis and treatments [1], its implementation has been left lagging behind, as several barriers have emerged. Among these, within the scientific community, there is ample debate as to whether the theoretical results proposed by research in this area can really be transformed into clinical practice, and as to whether healthcare professionals and citizens can fully understand the potential of these tools [2, 3].

In particular, health care professionals, in determining services to be offered to citizens or patients, might impede such implementation, with their limited knowledge in explaining the objectives and results of PM technologies and tools [4–8].

As a consequence, people are not appropriately aware of their benefits, risks, and clinical utility, and do not make appropriate health decisions. Therefore, the need to foster health literacy emerges, enabling them to access, understand and apply health knowledge, and to commit this knowledge into action, to make decisions regarding healthcare, disease prevention, and health promotion [9, 10].

The “Integrating China in the International Consortium for Personalised Medicine” (IC2PerMed) project is a Coordination and Support Action under the umbrella of the International Consortium of PM, that aims at providing guidance to overcome the abovementioned PM-related barriers in the European (EU) and Chinese contexts, so as to enable EU-Chinese collaborations for a sustainable healthcare system.

In order to recognise the relevance of the roles of healthcare professionals and citizens in PM, a mapping exercise of PM policies and initiatives in the EU and in China has been conducted from IC2PerMed [11–13]. The mapping results were subsequently used to list guiding issues that were presented during an expert workshop aimed at identifying a set of priorities in PM on healthcare professionals’ education and curricula, engagement and empowerment of citizens and patients.

## Materials and methods

### Selection of experts

In order to identify priorities to be addressed in PM, three online workshops were organised in the framework of the IC2PerMed project. The selection of topics discussed during the different workshops originates from an analysis of the key priorities listed in the ‘ICPerMed Vision for 2030’ together with the most relevant results of a mapping exercise concerning PM policies and initiatives in Europe and China. The mapping results are extensively reported in the Deliverable 1.1 available on the IC2PerMed website [13, 14]. One of the workshops explored the relevant guiding issues regarding healthcare professionals’ education and citizens’ engagement in PM, in the EU and in China. European and Chinese experts to be invited were identified in late 2020 using a bottom-up and top-down approach, that eventually resulted in involving 47 experts from the EU and 10 from China. Two coordinators of the working group (one from the EU and one from China) were identified among IC2perMed partners, to support and moderate discussion during the workshop. A list of guiding issues on the topic was prepared on the mapping exercise of European and Chinese policies in PM and distributed among the participants one week before the workshop [11–13]. The workshop dedicated to citizens’ awareness and empowerment and healthcare professionals’ education and curricula in PM was participated by 19 experts (16 from the EU and 3 from China).

### Delphi survey design

Preliminary priorities identified were then validated through a two-round consensus-based Delphi survey of experts. The survey included 28 items (4 questions on demographic information, 13 questions on education and curricula of healthcare professionals and 11 questions on awareness and empowerment of citizens and patients), divided into two sections:

- Sect. 1 with demographic information,
- Sect. 2 with the items of interest.

International experts with extensive experience in the field were invited to review the content of the items and to rate each one in terms of its validity and relevance by a five-point Likert scale, where 1 = strongly disagree and 5 = strongly agree. Experts also proposed additional priorities, therefore at the end of the first round, the survey was modified integrating feedback from all and then sent again to the panel for the second round of consultation. The entire procedure was conducted anonymously and disclosure of conflicts of interest was requested from the participants.

### Statistical analysis

For each item, according to the Delphi methodology, the Content Validity Index (CVI) was calculated [15, 16]. CVI ranges from 0 to 1, or from 0 to 100%, and results from the ratio between the number of experts that rate a singular item with 4 and 5 and the total number of experts involved. A CVI greater than 79% was deemed to be suggestive of item inclusion, a rate between 70 and 79% was considered indicative of the item revision, and a rate lower than 70% was deemed to be suggestive of item removal.

## Results

**Fig. 1:**
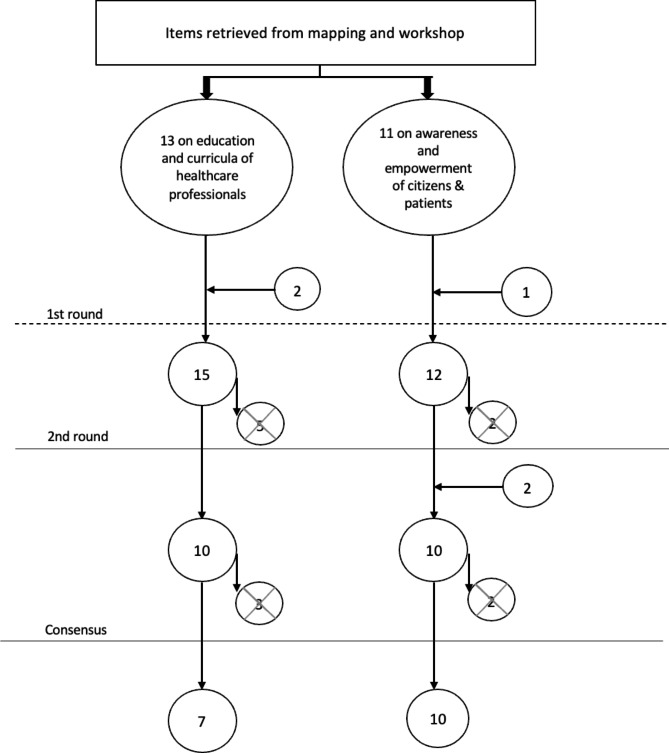
Flowchart of the Delphi survey process. The vertical flow indicates the number of priorities stemming before and after each Delphi survey round. The circles containing an X mark the number of priorities eliminated after each Delphi survey round. Horizontal arrows refer to priorities that were included following experts’ suggestions or any significant inputs from the workshops

Nine experts completed all the rounds (5 from the EU, 4 from China), representative of both the academic and clinical worlds. For the “education and curricula of healthcare professionals” topic, starting from 13 items retrieved from the workshops and activities of the project, two items were added, and five items were excluded during the first round. Experts reached a consensus on seven priorities, after excluding three items during the second round. For the “awareness and empowerment of citizens and patients” topic, two items were excluded, and one item was added to the initial 11 items in the first round. During the second round, experts suggested the addition of two items and the removal of the other two. In Fig. 1, the flowchart of the Delphi process depicts the results of each step.

Finally, seven priorities regarding health professional education and curricula and ten priorities regarding citizens’ and patients’ awareness and empowerment in PM were defined and reported separately below (Table 1).

**Table 1:** IC2PerMed priorities on “Healthcare Professionals’ Education and Curricula” and “Awareness and Empowerment of Citizens and Patients”

***IC2PerMed Priorities on “Healthcare Professionals’ Education and Curricula”***
1. Foster research aimed at identifying the most effective methods to improve healthcare professionals’ literacy and expertise in the field of PM
2. Strengthen healthcare professionals’ ethics
3. Improve healthcare professionals’ knowledge of ethical, legal, social, and economic issues related to PM
4. Improve healthcare professionals’ communication skills to inform and empower citizens and patients in the field of PM
5. Foster multidisciplinary collaborations between different healthcare professionals and stakeholders in PM-related practices
6. Establish collaborations/partnerships among institutions/countries aiming at improving healthcare professionals’ literacy and expertise in the field of PM
7. Include healthcare professionals’ health literacy in PM as an emerging priority in national governmental strategies, policies, and plans
***IC2PerMed Priorities on “Awareness and Empowerment of Citizens and Patients”***
1. Promote and support citizens’/patients’ engagement in self-management of health and disease
2. Improve populations’ health literacy and skills as a prerequisite for better citizens’ and patients’ engagement and empowerment in the field of PM
3. Improve citizens’ and patients’ digital literacy and skills, considering the role of digital tools in supporting the engagement of citizens in PM
4. Foster needs-assessment research in the field of citizens’ and patients’ education related to PM
5. Foster research activities aimed at identifying effective methods to improve citizens’ and patients’ literacy and engagement in the field of PM
6. Improve healthcare professionals’ genomic literacy as a prerequisite for effective citizens’ and patients’ engagement in the field of PM
7. Strengthen communication and enhance communication activities in the field of PM
8. Promote public trust in scientific research, public organizations, and private institutions, given their importance in advancing PM-related research and technology
9. Foster collaborations/partnerships between different institutions/countries aiming to improve citizens’ and patients’ literacy and empowerment in the field of PM
10. Consider ethical, legal, social, and economic challenges for citizens’ and patients’ engagement in the field of PM

The organisation of the results used in this article involves the presentation of the priority according to the exact phrasing obtained through the Delphi consultation and a brief discussion of the key elements presented by the experts regarding the specific priority a throughout the consultations.

### Priorities regarding healthcare professionals’ education and curricula

In order to enhance healthcare workforce skills, updating training programs and educational curricula has emerged as a major need for healthcare professionals to be up-to-date with the latest findings. So, in this regard, the curricula of healthcare professionals should be updated and adapted for the most appropriate usage of PM tools, which has been widely recognized as both important and challenging [17].

From this perspective, despite the wide recognition of the importance of knowledge in this field, the perception of many health professionals is that they have not received adequate education about it. Therefore, the development of educational programs specifically aimed at bridging this gap seems to be essential [18].

Priorities identified from this study are consistent with the needs discussed so far and support the importance of efforts in improving the curricula of health professionals from many perspectives: health literacy, ethics, legal, and economic components, as well as communication skills and multi-professional collaboration.

**Priority 1: Foster research aimed at identifying the most effective methods to improve healthcare professionals’ literacy and expertise.**

Appropriate and validated tools should be adopted to ensure rapid and effective integration of new discoveries into professionals’ education and curricula. Investments in research capacities and infrastructure, for the development of such tools, should be among the priorities of both European and Chinese policy agendas. In addition, effectiveness of PM-related research and advocacy should also be ensured and measured.

**Priority 2: Include healthcare professionals’ health literacy in PM as an emerging priority in national governmental strategies, policies, and plans.**

National government strategies, policies, and plans addressing health worker literacy should address all relevant PM issues, including opportunities brought by innovative technologies and research, as well as the operation of information and data systems, and financial frameworks. Therefore, healthcare professionals should be knowledgeable about economic aspects, as well as ethical, legal, and social implications (ELSI), in order to support and promote quality and sustainability in clinical practice and research. The role of governments and government agencies in promoting this type of improvement in the training provision for health professionals in the field of personalised medicine is essential. Only centrally coordinated action within the framework of training programmes and periodic skill-upgrading cycles can have a real impact on the training of health professionals in this field. In addition to providing up-to-date information and guidance, health literacy programs could implement risk communication and disease management, so that health professionals would be able to engage patients through informed consent and decisions. Non-technical skills should be categorized and formally addressed in policies and plans as technical skills.

**Priority 3: Strengthen healthcare professionals’ ethics.**

The recent development of PM, with the advancement of genomic sequencing methods, the growth of artificial intelligence and the increasing demand for data have opened the way, on one hand to exciting clinical and therapeutic prospects, but on the other hand to numerous ethical issues [19]. These issues develop on a highly multidisciplinary ground, moving between the potential lack of health literacy for obtaining informed consent, incidental findings in genetic testing, and the possibility of changing the patient-physician relationship by focusing on data, and many others [20–22].

Therefore, strengthening healthcare professionals’ integrity and ethics could provide a useful instrument that might limit the extension of these risks. Informed management of resources within PM by health professionals can provide greater patient protection by helping to promote fruitful collaborations for population health outcomes.

**Priority 4: Improve healthcare professionals’ knowledge of ethical, legal, social, and economic issues.**

A wide range of ELSI issues has been related to the implementation of PM methods in health care systems. The proper use of informed consent to protect the health data of patients and their families, possible social discrimination in access to PM tools, and potential implications in access to insurance coverage depending on the results of genetic testing are just some of the issues to be paid attention to in implementation in this area [23].

In this complex scenario, training of health professionals might mitigate such issues, by creating a workforce skilled and attentive enough to manage health data and information derived from new methods in the most profitable and respectful way of patients’ preferences.

**Priority 5: Improve healthcare professionals’ communication skills to inform and empower citizens and patients.**

One of the elements that affects the accessibility of care in the PM sector, and ultimately the health of the patients themselves, is the communication skills of healthcare professionals [24].

While patients’ understanding of health information is influenced by the level of health literacy of the individual receiving the information, it is equally true that the ability of the health professional to convey the information effectively is nonetheless important [25].

Communication skills, while not exactly part of a skill set traditionally addressed in academia, are also rightfully among the elements that need to be integrated into the curriculum of health care professionals, and therefore there is a need to pay attention to these elements within the educational and continuing education plans of professionals who deal with patients on a daily basis. In this sense, the adoption of methods aimed at promoting effective communication, such as Active Listening and Teach Back, advances an interesting perspective for integrating a greater understanding of patient needs with the effective conveyance of health information by the health professional [26, 27]. For these reasons, the study of effective methods to promote training in this area of health communication has been identified as a critical priority, both in Chinese and European contexts.

**Priority 6: Foster multidisciplinary collaborations between different healthcare professionals and stakeholders.**

In order to obtain the most beneficial result in terms of patient health, collaborations between healthcare professionals specialized in different fields are essential to setting up an appropriate apparatus of skills and professionalism, extended to all areas of PM. However, a wider collaboration at the international level might help to overcome the fragmented landscape of PM across different countries, and to achieve the best implementation of PM in the healthcare system while valuing different perspectives. On a broader level, multidisciplinary collaborations should be promoted in PM-related practices, as they have proven to be more effective.

The role of education of health professionals in multidisciplinary collaboration is central. Indeed, the goal of promoting this kind of inclusive approach that is open to other disciplines passes through a mindset that has as its starting point the propensity of practitioners and researchers to collaborate; this attitude can only be nurtured by training programs that include these elements from the earliest stages of approach to scientific research as much as to the clinical setting.

**Priority 7: Establish collaborations/partnerships among institutions/countries aiming at improving healthcare professionals’ literacy and expertise.**

Currently, there is a huge lack of standardization and uniformity when providing health care related to PM, both at the European and Chinese levels. This regulation gap consequently complicates the identification of an internationally shared set of skills and knowledge with reference to the literacy of healthcare professionals. While creating disparities across different settings, it poses the need to identify key players who can fit into a multicultural and bilateral dialogue aimed at developing a framework to unify policies, skills and competencies for PM. Universities, research organizations, and national and international institutions should have a central role in this process, aiming at the creation of a commonly shared point of view. Programs that promote national and international consortia or partnerships that aim to standardize the framework on PM should be prioritized.

### Priorities regarding awareness and empowerment of citizens and patients

Active involvement of citizens and patients in the use of PM tools is essential for their proper implementation, and this comes through knowledge that enables their informed use, especially considering that personalised approaches support the identification of relevant self-management interventions based on well-characterized phenotypes, such as genetic, behavioural, environmental, and physiological [28].

**Priority 1: Promote and support citizens’/patients’ engagement in self-management of health and disease.**

Adequate health literacy helps individuals become more aware of their health and improve their ability to make well-informed decisions. Increasing and improving self-management therefore requires better communication between professionals and patients and effective, targeted initiatives to increase public engagement and knowledge. Promoting citizens’ health literacy can increase their ability to make informed health choices and proactively self-manage by engaging in the doctor-patient relationship.

**Priority 2: Improve populations’ health literacy and skills as a prerequisite for better citizens’ and patients’ engagement and empowerment.**

PM requires citizens to have better knowledge and greater involvement, enabling them to use the right tools to actively participate in the improvement of the health system, with better awareness and decision-making power. Health literacy represents the first step in the process of empowerment and participation of citizens, according to International Association for Public Participation (IAP2) [29]. This process could lead to the development of a virtuous circle, in which the patient centred-care feeds (with its data) into patient entered-research, which in turn provides data to get patient-oriented evidence, to obtain a full patient engagement in clinical research, and, finally, a disease-oriented and patient-oriented outcome and create a better patient centred-care. This loop can provide the proper assessment of health literacy to improve the engagement of patients and citizens.

**Priority 3: Improve citizens’ and patients’ digital literacy and skills, considering the role of digital tools in supporting citizens’ engagement.**

To improve population health, digital health is a necessary tool and is increasingly being integrated into the health care system. The role of health literacy in this area is focal to enable citizens to use these tools correctly and ultimately to derive the greatest possible health benefits from them, so it is necessary to develop interventions aimed at helping healthcare professionals and patients familiarize themselves through the dissemination of digital tools. The progressive digitalization of modern societies, and healthcare, requires digital literacy and skills to be implemented. However, despite the great progress in digital health in recent years, the public’s knowledge is still inadequate even in the most industrialized countries.

**Priority 4: Foster needs-assessment research in the field of citizens’ and patients’ education related to PM.**

Public involvement in PM is critical so that governments can develop policies based not only on research and expert opinion but also on the experiences of citizens and patients. Through needs-assessment research it might be possible to understand what the population’s unmet needs are, especially in terms of health literacy, and to make targeted changes and improvements. Even though it is the first step of intervention mapping [30], which is the main framework for bridging theory and practice, it is often neglected for the scarcity of funding or for low economic interests.

**Priority 5: Foster research activities aimed at identifying effective methods to improve citizens’ and patients’ literacy and engagement.**

Despite the introduction of numerous tools to improve health literacy and engagement of citizens and patients in PM (including new technologies and digital services, education courses, workshops, and webinars), it is difficult to evaluate their effectiveness and determine which of them should be used. Therefore, investing in research to identify the most effective teaching and education approaches for citizens and patients is crucial to enable them to benefit from the implementation of PM in the healthcare system.

**Priority 6: Improve healthcare professionals’ genomic literacy as a prerequisite for effective citizens’ and patients’ engagement in the field of PM.**

To implement citizens’ genomic literacy, one of the main tools is face-to-face discussions with healthcare professionals [31], given their close communication with citizens and patients. Genomics is one of the main pillars of PM, and its importance has been emphasized for rare diseases and chronic diseases, such as cancer and cardiovascular disease, to the development and adoption of polygenic risk scores. Genomic literacy of health professionals should be implemented, enhancing the effectiveness of the tools employed [32]. With the spread of DTC-GTs, it is of paramount importance that health care providers are able to answer citizens’ questions and establish effective communication in the doctor-patient relationship.

**Priority 7: Strengthen communication and enhance communication activities.**

For increased public awareness, communication should be managed both at the population level, with public and private institutions, and at the level of the health system, between healthcare professionals and patients and between healthcare professionals of different specialties. International collaborations should be nurtured, in order to promote the sharing of knowledge, ideas and experiences, that might improve citizens’ knowledge and understanding.

**Priority 8: Promote public trust in scientific research, public organizations, and private institutions, given their importance in advancing PM-related research and technology.**

Public trust facilitates the adoption of new policies, processes, and technologies, especially in health care. Therefore, ensuring transparency in decision-making and promoting dialogue among parties, whether in research, public organizations or private institutions, can help strengthen public trust in health systems and PM. Networking and transparency in scope are two key factors to sustain research and both public and private institutions [33, 34]. The success of PM depends on the use of huge amounts of data (big data), related to genomic and health information. Public trust in the ability of the government and health system to securely store, disseminate, and analyse data is a crucial factor in the implementation of PM practices [35].

**Priority 9: Foster collaborations/partnerships between different institutions/countries aiming to improve citizens’ and patients’ literacy and empowerment.**

Institutions and governments can have a significant role in promoting PM and public health, and their collaborations and partnerships could help share best practices and overcome national barriers related to different healthcare systems and political and cultural frameworks.

**Priority 10: Consider ethical, legal, social, and economic challenges for citizens’ and patients’ engagement.**

Citizens’ and patients’ engagement poses numerous ELSI and economic challenges that affect all stakeholders. The lack of knowledge of omics technologies, coupled with the need to use personal patient data, makes citizens often hesitant to get involved in clinical applications of PM and even more so in studies testing its effectiveness. Considering its complexity and the heterogeneity of potential beneficiaries, it is fundamental to ensure both tangible and intangible means of all forms (e.g., necessary education, digital infrastructure, availability of financial resources), to benefit from PM practices.

## Discussion

The implementation of PM in health systems is appealing in terms of improved accuracy in diagnostics, treatment, and prevention of disease, as well as the reduction of side effects resulting from inappropriate use of treatments [36]. Such positive results on the clinical side are even more appealing if combined with an improved use of resources that a more adequate targeting of medical interventions and resources could establish. These benefits are derived from scientific advancement of medicine and new knowledge, which in turn lead to better, more targeted approaches and safer interventions. However, the existence of new and more advanced tools does not necessarily have the same level of availability and accessibility for researchers, citizens and patients, health professionals and other stakeholders in the field [37]. In our workflow, we performed a mapping of the available policies, initiatives and evidence, trying to bring to light the limitations and barriers in this area [13]. As previously highlighted, the results of this mapping, together with the direction identified by the ICPerMed consortium, served as a starting point for the identification of a set of priorities. Priorities regarding healthcare professionals’ education and curricula and citizen empowerment are here reported and discussed. The importance of these priorities lies in providing a common starting point, shared from both a European and a Chinese perspective, for targeted and potentially synergistic action on the development of PM.

From a professional healthcare perspective, insufficient health literacy, especially the PM related advancing health literacy might lead to difficulties in interpreting omics-based diagnostic tests (e.g., disease-specific biomarkers) as well as the underappreciation of benefits deriving from an available therapy (e.g., advanced cancer treatments targeting specific antigens). When healthcare professionals are lacking these capabilities, it is more likely that they will take ill-considered decisions, discouraging the use of means of which they do not have full mastery, and maybe even poorly communicate the clinical results to patients [38].

On the patient side, poor understanding of the disease and of available interventions deriving from scarce health literacy is related to lacking medical adherence, unsatisfying compliance to medical therapies, and a poor physician-patient relationship [39].

People with poor health literacy could be less likely to ask clarifying questions because of embarrassment or fear, which can lead to an inaccurate treatment or diagnosis [40]. Consequently, they are generally 1.5–3 times more likely to experience negative health outcomes [41]. This situation has relevant repercussions on the quality of the dialogue between patients, their families, and healthcare professionals. A relevant consequence of low literacy is actually the inability to make effective use of healthcare services, leading to a poorer ability to self-manage a health condition and to fewer adherences to treatment plans [42]. Despite enormous advancements in digital technology and health information technology, the general population is still not adequately informed and involved in PM [43, 44]. Many factors can contribute to the digital divide, worsening health outcomes for some populations as digital tools become more widespread [45, 46]. For this reason, it is important to focus efforts on promoting digital health literacy to empower the population and improve citizens’ engagement in PM.

Successful implementation of PM also requires close attention to ELSI aspects and challenges arising from research into the population needs. Since PM is an extremely complex and varied field in terms of possibilities, both at the level of individual and society, it becomes necessary for policymakers to ensure the necessary resources and expertise at every level. In several surveys, citizens expressed very limited knowledge, a strong interest in receiving further education, and concerns about data sharing and ELSI implications [47].

The lack of clear rules addressing data management and the healthcare professional-patient relationship brought universities and national institutions to pursue different visions of concepts related to PM between different educational providers. It is therefore necessary to prioritize programs promoting national and international consortia, aiming at standardising the educational framework on PM. Within the ELSI aspects of PM, ensuring transparency in processes and decisions and fostering dialogue between parties have important repercussions on public trust. Public trust is based on the reliability and accountability of the information source or services and ensures the commitment of both parties to achieving an outcome [48]. Consequently, where public trust is present, policies and changes are more readily adopted, facilitating the advancement of PM research and practices [49].

Collaboration between all the actors involved in the field should take place not only transversally within the health system, through the physician-patient relationship, but at all levels: starting with the citizens and moving to health professionals of different medical specialties, industries, academies and, finally, at national and international governmental level. In this context, policies are fundamental in achieving the goals of awareness and empowerment of citizens and patients, improving the curricula and education of health professionals, and enabling the development of initiatives aimed at national and international cooperation. For this reason, national and international government strategies, policies, and plans addressing healthcare professionals’ literacy should be on top of the agenda to promote quality and sustainability in clinical practice and research. Without this communication effort among the healthcare system, institutions, and citizens, the general population will have reduced capacity to take decisions about their care and may be wary of the broader tools of PM.

The purpose of this work was to lay the foundations for tailoring policy design to the actual needs that emerged from the identified priorities. Filling a gap in the scientific literature on the most relevant areas to be dealt with in PM, our work provides, to our knowledge, an international overview of healthcare professionals’ and citizens’ standpoints for PM implementation.

Priorities identified in this study are consistent with the challenges proposed by ICPerMed and support the importance of efforts in improving the literacy and skills of citizens and health professionals from many perspectives.

Despite the strict methodology, this work should be considered in light of some limitations. The resulting priorities cover many topics, but more issues to be investigated might have been neglected, likely because of the inaccessibility of relevant documents on the web, or participants’ and experts’ own cultural and work backgrounds. In addition, bringing together European and Chinese perspectives could lack useful perspectives from participants elsewhere.

## Conclusions

PM is a complex and multidisciplinary field, and research must be oriented in the best possible way to ensure it reaches its goals. Over the last few years, there has been a lot of emphasis on this field and great attention has been focused on educating health professionals and involving the population in the issues of prevention and care, established in a personalised approach.

Addressing and improving citizens’ literacy and attitudes have been considered strategic factors to increase people’s control over their health, their ability to take responsibility for lifestyle choices and seek out health information, leading to the promotion and protection of health and prevention of diseases, therefore improving the whole population’s health and well-being [50]. However, despite efforts to improve the population’s PM knowledge, there is still a need for fostering research activity aimed at identifying effective ways to improve citizens’ and patients’ PM literacy and engagement.

To accomplish this task, it is also crucial that healthcare professionals are continually informed and trained in the latest innovations in medical sciences and the dissemination process, through education programs and the use of innovative and digital tools. Policies addressing this issue should focus not only on education programs, but also on addressing socio-economic inequalities, addressing the different inequalities. The collaboration between different stakeholders at all levels of healthcare is fundamental to achieving the best standard of care. A health alliance between health professionals, citizens, and policymakers should be established, aiming at integrating perspectives and needs, and working together to design PM in a way that can be effectively adopted.

## Data Availability

All the documents mentioned in the manuscript and the workshops’ results are available for consultation at https://www.ic2permed.eu/zh/publications-public-deliverables-2/.

## Abbreviations

ELSI: Ethical, Legal, Social Issues
EU: European Union
ICPerMed: International Consortium for Personalised Medicine
IC2PerMed: Integrating China in the International Consortium for Personalised Medicine
PM: Personalised Medicine
